# Age- and Sex-Specific Differences in Lyme Disease Health-Related Behaviors, Ontario, Canada, 2015–2022

**DOI:** 10.3201/eid3010.240191

**Published:** 2024-10

**Authors:** Janica A. Adams, Victoria Osasah, Katherine Paphitis, Affan Danish, Richard G. Mather, Curtis A. Russell, Jennifer Pritchard, Mark P. Nelder

**Affiliations:** Public Health Ontario, Toronto, Ontario, Canada (J.A. Adams, V. Osasah, K. Paphitis, A. Danish, R.G. Mather, C.A. Russell, J. Pritchard, M.P. Nelder);; Johns Hopkins Bloomberg School of Public Health, Baltimore, Maryland, USA (V. Osasah);; Queen’s University, Kingston, Ontario, Canada (R.G. Mather)

**Keywords:** Lyme disease, vector-borne infections, zoonoses, *Borrelia burgdorferi*, blacklegged ticks, bacteria, epidemiology, *Ixodes scapularis*, ticks, tickborne diseases, surveillance, One Health, Canada

## Abstract

We investigated differences in risk factors and preventive behaviors by age and sex among persons with reported Lyme disease in Ontario, Canada, during 2015–2022. Incidence rates peaked among children 5–9 and adults 50–79 years of age. Median age was higher for female than male case-patients (54 vs. 51 years). Male case-patients reported more activity in wooded and tall grass areas than did female case-patients; fewer male case-patients reported sharing living space with outdoor-exposed companion animals. As age increased, more case-patients reported activity in blacklegged tick habitats, exposure to ticks, and wearing adequate clothing, but fewer reported sharing living space with outdoor-exposed companion animals. Adoption of preventive behaviors was relatively low and did not differ by sex. Male case-patients, children 5–9 years of age and their parents or caregivers, and adults >59 years of age represent populations that would benefit from tailored public health messaging on Lyme disease prevention.

Lyme disease is the most reported vectorborne disease affecting humans in Canada, and incidence continues to increase (*1*). In eastern North America, the blacklegged tick (*Ixodes scapularis*) is the primary vector of *Borrelia burgdorferi* sensu stricto, the causative agent of Lyme disease. About half of all cases of Lyme disease in Canada are reported from Ontario, a province in which blacklegged tick range has rapidly expanded northward by ≈50 km/year (*2*–*5*). *I. scapularis* ticks were first detected in Ontario along the northern shore of Lake Erie in the early 1970s (*6*). From the 1970s through the 2000s, blacklegged tick populations remained relatively isolated along the northern shores of the St. Lawrence River, Lake Ontario, and Lake Erie; however, in the 2010s, populations expanded or were established anew throughout the province (*7*). Climate and land-use changes will continue influencing the expansion of blacklegged tick range, increasing the risk for Lyme disease (*8*–*10*). 

Blacklegged ticks transmit pathogens in addition to *B*. *burgdorferi* to humans, including *Anaplasma phagocytophilum*, *B*. *mayonii*, *B*. *miyamotoi*, *Babesia microti*, Powassan virus (lineage 2, or deer tick virus), and *Ehrlichia muris eauclairensis* (*11*). In 2023, postexposure prophylaxis was made available to residents of Ontario as part of a new Lyme disease prevention option, enabling pharmacists to treat patients after assessing their symptoms and tick exposure history (*12*). Optimizing public health guidance for preventing Lyme disease requires approaches focused on minimizing exposure to blacklegged ticks. 

Our goal was to examine sex- and age-specific differences in exposures to Lyme disease risk factors and preventive behaviors adopted, which might increase or decrease risk of exposure to blacklegged ticks and local transmission of Lyme disease in Ontario compared with neighboring jurisdictions (e.g., the province of Quebec, as well as the continental United States, including New Jersey and New York) (*13*–*17*). Because climate and land-use changes affect the expansion of ranges among ticks, awareness of population subgroups experiencing increased rates of illness can help inform evidence-based public health messaging. We performed a retrospective cross-sectional study, using provincially reportable Lyme disease data to examine age- and sex-specific differences in self-reported risk factors and preventive behaviors associated with Lyme disease in Ontario during 2015–2022. 

This project did not require research ethics committee approval because the activities were considered public health surveillance. Those activities were conducted in fulfillment of a Public Health Ontario legislative mandate “to provide scientific and technical advice and support to the health care system and the Government of Ontario in order to protect and promote the health of Ontarians.” (Ontario Agency for Health Protection and Promotion Act, SO 2007, c 10, Schedule K). 

## Methods

### Surveillance

In Ontario, 34 local public health units (PHUs) are responsible for the surveillance, investigation, and management of Lyme disease cases and also take part in active (dragging) and passive (identification of publicly submitted ticks) tick surveillance (*18*,*19*). PHUs classify confirmed and probable cases of Lyme disease on the basis of current provincial case definitions (Appendix Table). By telephone interview, investigators from the PHU area within which the case-patient lived at time of illness collected information using the provincial Lyme disease investigation tool, a standardized questionnaire for Lyme disease (*20*). Case information collected included likely location of tick bite (e.g., cottage, house, park), risk factors (e.g., exposure to ticks, activities in wooded areas), and preventive behaviors (e.g., checking self for ticks, wearing appropriate clothing, using tick repellents) associated with decreased risk for Lyme disease infection. Case-patients who reported health behaviors that potentially increased risk for developing Lyme disease during the telephone interview were asked about the most likely location of tick exposure (*7*) and might have voluntarily provided additional information on exposures, risk factors, and preventive behaviors. Additional information collected from all case-patients included age, sex/gender (male, female, transgender, other, or unknown), and month and year of episode. Case-patient demographic and exposure data were reported to provincial public health authorities through the integrated Public Health Information System, an online surveillance platform. 

### Statistical Analyses

We conducted a descriptive analysis of Lyme disease risk factors and preventive behaviors to explore sex- and age-specific differences among case-patients. We recorded month and year of episode using, in order of preference, time of onset, specimen collection, laboratory testing, or case reported. We used patient’s age at date of episode. We categorized case-patients >10 years of age into 10-year age groups (e.g., 10–19, 20–29). However, we determined 5-year age groups for patients <10 years of age because some previous literature suggested a relatively higher incidence of Lyme disease among children 5–9 years of age than children of other ages (*14*). To calculate average incidence rates per 100,000 persons, we used Government of Canada annual population estimates for Ontario (*21*). 

For analyses involving sex as a variable, we included only case-patients who self-identified as male or female because of low counts (n<5) for other sex/gender categories. We compared demographic characteristics of male and female case-patients across age groups. For analyses involving geographic location, we included only cases with reported exposures within Ontario; we excluded from subsequent analyses all case data from persons reporting travel outside of Ontario during the exposure period. We used the Mann-Whitney U test to assess differences in medians, the Pearson χ^2^ test to compare risk factors reported by male and female case-patients, and the Cochran-Mantel-Haenszel test to assess associations between age group and sex and between age group and self-reported risk factors. We performed all analyses, data cleaning and classification of exposures, risk factors, and preventive behaviors in Microsoft Excel (Microsoft, https://www.microsoft.com) and SAS Enterprise Guide 8.2 (SAS Institute Inc., https://www.sas.com). We considered differences among variables to be statistically significant at p<0.05. 

## Results 

### Epidemiology

**Figure 1:**
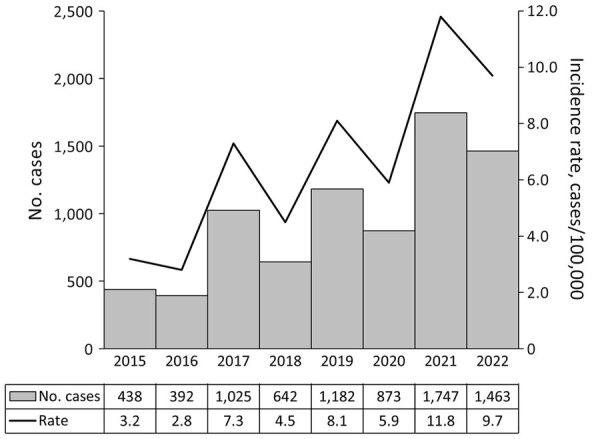
Annual case counts and incidence rates for Lyme disease in Ontario, Canada, 2015–2022. Denominators used in rate calculations based on annual provincial population estimates.

During 2015–2022, there were 7,762 cases of Lyme disease reported in Ontario; 7,213 (92.9%) cases were confirmed and 549 (7.1%) probable (Table 1; Figure 1). Incidence increased 3-fold during the study period. Annually, the highest proportion of cases occurred during the summer, from June through August (Appendix Figure 1). 

**Table 1:** Demographics of Lyme disease case-patients in Ontario, Canada, 2015–2022*

Demographics	Value	Average annual incidence rate†
Age, y, n = 7,758‡		
Median (IQR)	53 (32–64)	NA
Range	<1–95	NA
Age group, y, n = 7,762		
<5	180 (2.3)	3.1
5–9	440 (5.7)	7.2
10–19	557 (7.2)	4.3
20–29	567 (7.3)	3.5
30–39	733 (9.5)	4.6
40–49	975 (12.6)	6.5
50–59	1,497 (19.3)	9.2
60–69	1,636 (21.1)	11.8
70–79	954 (12.3)	10.8
≥80	219 (2.8)	4.2
Unknown	4 (<0.5)	NA
Sex/gender, n = 7,762§		
M	4,371 (56.3)	7.6
F	3,364 (43.3)	5.7
Transgender/other/unknown	27 (<0.5)	NA

*Values are no. (%) patients except as indicated. IQR, interquartile range; NA, not available †Rates per 100,000 persons were calculated for each age group using age-stratified annual population estimates in Ontario and for each sex using sex-stratified annual population estimates in Ontario. ‡Data for age were unavailable for 4 case-patients. Analysis was restricted to case-patients with complete data for age. §Analysis was restricted to case-patients who self-identified as male or female because of low counts (n<5) observed in other reported categories.

**Figure 2:**
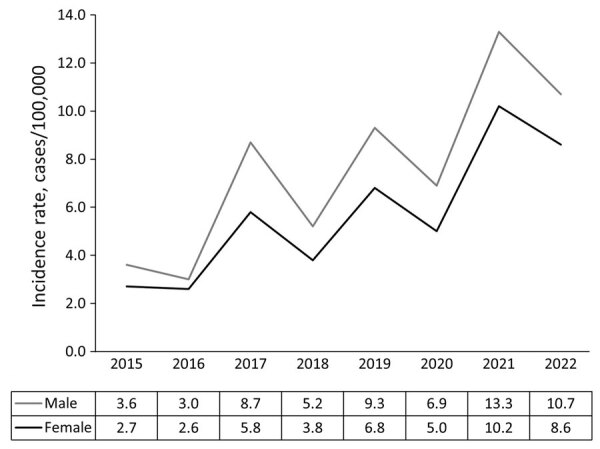
Annual incidence rates of Lyme disease case-patients, by sex, Ontario, Canada, 2015–2022. Denominators used in rate calculations include annual population estimates stratified by sex.

Twenty-seven case-patients entered sex as other than male or female (27/7,762, 0.3%), 3 as transgender or other and 24 as unknown; because of low numbers, we excluded those 27 case-patients from analyses involving sex. In addition, we excluded 4 case-patients with no data for age from analyses involving age. The median age of case-patients was 53.0 years; the most common age groups were 60–69 years (1,636/7,758 [21.1%]) and 50–59 years (1,497/7,758 [19.3%]) (Table 1). By sex, 56.3% of case-patients were male and 43.3% female; <5% were categorized as transgender, other, or unknown (Table 1). Annual Lyme disease incidence rates were higher among male than female case-patients (Table 2; Figure 2). Median age was lower for male than female case-patients (51.0 years vs. 54.0 years; χ^2^ = 16.08; p<0.001). Average annual incidence rates per 100,000 persons peaked among children 5–9 years of age (7.2%) and adults in age groups 50–59 (9.2%), 60–69 (11.8%), and 70–79 (10.8%) years of age (Appendix Figure 2). Although the number of cases generally increased each year, overall annual rates did not differ significantly by sex or age group (p>0.05). 

**Table 2:** Demographics of Lyme disease case-patients, by sex, Ontario, Canada, 2015–2022*

Demographics	Total, n = 7,731	Female, n = 3,362	Male, n = 4,369	Test of association	p value
Age, y					
Median (IQR)	53.0 (32.0–64.0)	54.0 (35.0–65.0)	51.0 (31.0–64.0)	16.08†	<0.001
Range	<1–95	<1–94	<1–95		
Age group, y					
<5	180 (2.3)	81 (45.0)	99 (55.0)	16.89‡	<0.001
5–9	438 (5.7)	191 (43.6)	247 (56.4)		
10–19	556 (7.2)	212 (38.1)	344 (61.9)		
20–29	565 (7.3)	212 (37.5)	353 (62.5)		
30–39	730 (9.4)	300 (41.1)	430 (58.9)		
40–49	972 (12.6)	382 (39.3)	590 (60.7)		
50–59	1,488 (19.2)	683 (45.9)	805 (54.1)		
60–69	1,633 (21.1)	791 (48.4)	842 (51.6)		
70–79	950 (12.3)	409 (43.1)	541 (57.0)		
≥80	219 (2.8)	101 (46.1)	118 (53.9)		

*Values are no. (%) patients except as indicated. IQR, interquartile range. †Mann-Whitney U test; p<0.05 represents a statistically significant difference in the median age of female and male case-patients. ‡Cochran-Mantel-Haenszel test; p<0.05 represents a statistically significant relationship between increasing age groups and sex.

### Exposures, Risk Factors, and Preventive Behaviors

A total of 4,108 case-patients voluntarily reported >1 specific exposure activities (results not shown); interviewers using the Ontario Lyme disease questionnaire did not routinely ask case-patients about specific exposures. Among common exposure activities, 1,891/4,108 (46.0%) case-patients providing responses reported visiting a secondary residence, such as a cottage; 1,264 (30.8%) reported exposure to a tick habitat in the primary residence or when visiting a friend or relative’s home, and 474 (11.5%) reported visiting a park. Less commonly reported activities included camping (170/4,108 [4.1%]), visiting a hiking or cycling trail (150 [3.7%]), and visiting a conservation area (127 [3.1%]). 

Interviewers using the Ontario Lyme disease questionnaire asked all case-patient respondents about risk factors and preventive behaviors. Of case-patients providing (yes/no) responses, 81% (6,289/7,762) reported >1 risk factor or preventive behavior; ≈89% (4,430/4,937) of case-patients provided responses to >1 question about exposure to an at-risk area for Lyme disease, tick habitat, or reported activities in blacklegged tick habitats (wooded or tall grass areas); 4,020 (86.0%) recalled an exposure to ticks or having a tick bite; and 1,486 (41.9%) reported sharing a bed or indoor living space with a companion animal that had access to the outdoors (Table 3). 

**Table 3:** Specific risk factors and preventive behaviors reported by Lyme disease case-patients, by sex, Ontario, Canada, 2015–2022*

Exposure activities	No. (%) cases			Crude OR (95% CI)†	p value‡
	Total	Female	Male		
Risk factors					
Participated in activities in wooded or tall grass areas§					
N	507	282 (55.6)	225 (44.4)	1.77 (1.47–2.13)	<0.001
Y	4,430	1,835 (41.4)	2,595 (58.6)		
Recalled finding tick or tick bite on self					
N	652	301 (46.2)	351 (53.8)	1.18 (1.00–1.39)	0.05
Y	4,020	1,691 (42.1)	2,329 (57.9)		
Contacted outdoor dog or cat that shared bed or living space					
N	2,062	864 (41.9)	1,198 (58.1)	0.82 (0.72–0.94)	0.004
Y	1,486	696 (46.8)	790 (53.2)		
Preventive behaviors					
Checked for ticks after being outdoors in wooded or tall grass areas					
N	2,650	1,107 (41.8)	1,543 (58.2)	0.88 (0.77–1.01)	0.07
Y	1,265	567 (44.8)	698 (55.2)		
Used insect repellant when outdoors in wooded or tall grass areas					
N	2,805	1,187 (42.3)	1,618 (57.7)	0.89 (0.77–1.03)	0.12
Y	958	433 (45.2)	525 (54.8)		
Used adequate clothing protection in wooded or tall grass areas¶					
N	2,704	1,128 (41.7)	1,576 (58.3)	0.87 (0.76–1.01)	0.07
Y	1,046	471 (45.0)	575 (55.0)		

*Case-patients were queried on risk factors and preventive behaviors in the 30 d before illness onset. A low percentage of case-patients provided responses to queries on risk factors (46%–64%) and preventive behaviors (48%–50%). OR, odds ratio. †Reference group used in OR calculations is female case-patients. ‡χ^2^ test; p<0.05 represents a statistically significant relationship between risk factor/preventive behavior and sex. §For activities in wooded or tall grass areas, case-patients were asked about specific activities such as hiking, camping, or hunting. ¶Adequate clothing included long sleeves, long pants, and covered shoes.

Male case-patients had higher odds than female of reporting activities in blacklegged tick habitats (crude odds ratio [OR] 1.77, 95% CI 1.47–2.13; p<0.001) and lower odds of sharing living space with an outdoor companion animal (OR 0.82, 95% CI 0.72–0.94; p = 0.004). There was also a significant association between age and reported activities, and in general, as age increased, respondents reported more activities in blacklegged tick habitats (n = 4,948, degrees of freedom [df] = 1, χ^2^ = 15.69; p<0.001) and finding a tick on themselves (n = 4,680, df = 1, χ^2^ = 14.22; p<0.001) (Table 4). As age increased, fewer case-patients reported sharing living space with an outdoor companion animal (n = 3,555, df = 1, χ^2^ = 8.71; p = 0.003). 

**Table 4:** Risk factors and preventive behaviors reported by Lyme disease case-patients, by age group, Ontario, Canada, 2015–2022*

Exposure activities	No. (%) case-patients by age group, y											χ^2^†	p value
	Total	<5	5–9	10–19	20–29	30–39	40–49	50–59	60–69	70–79	≥80		
Risk factors													
Participated in activities in wooded or tall grass areas‡													
N	507 (10.2)	18 (15.1)	20 (6.5)	25 (6.7)	41 (10.9)	44 (8.8)	63 (9.8)	111 (11.4)	99 (9.8)	70 (13.3)	16 (14.6)	15.69	<0.001
Y	4,441 (89.8)	101 (84.9)	290 (93.6	346 (93.3)	336 (89.1)	458 (91.2)	581 (90.2)	863 (88.6)	915 (90.2)	457 (86.7)	94 (85.5)		
Recalled finding tick or tick bite on self													
N	653 (14.0)	18 (18.2)	47 (17.3)	60 (19.5)	47 (15.1)	53 (12.5)	106 (18.0)	127 (13.6)	118 (11.5)	63 (10.8)	14 (10.9)	14.22	<0.001
Y	4,027 (86.0)	81 (81.8)	224 (82.7)	248 (80.5)	265 (84.9)	371 (87.5)	483 (82.0)	806 (86.4)	912 (88.5)	522 (89.2)	115 (89.2)		
Contacted outdoor dog or cat that shared bed or living space													
N	2,066 (58.1)	53 (56.4)	121 (53.8)	154 (56.4)	168 (63.9)	173 (49.4)	273 (57.6)	376 (54.6)	442 (60.2)	243 (66.2)	63 (73.3)	8.71	0.003
Y	1,489 (41.9)	41 (43.6)	104 (46.2)	119 (43.6)	95 (36.1)	177 (50.6)	201 (42.4)	313 (45.4)	292 (39.8)	124 (33.8)	23 (26.7)		
Preventive behaviors													
Checked for ticks after being outdoors in wooded or tall grass areas													
N	2,657 (67.7)	62 (63.9)	170 (68.0)	209 (70.1)	211 (69.9)	266 (67.7)	354 (67.6)	506 (66.8)	528 (65.8)	278 (68.8)	73 (77.7)	0.46	0.50
Y	1,266 (32.3)	35 (36.1)	80 (32.0)	89 (29.9)	91 (30.1)	127 (32.3)	170 (32.4)	252 (33.3)	275 (34.3)	126 (31.2)	21 (22.3)		
Used insect repellant when outdoors in wooded or tall grass areas													
N	2,812 (74.6)	67 (72.8)	169 (70.1)	227 (79.7)	216 (74.2)	287 (75.5)	382 (76.1)	538 (74.0)	557 (72.0)	296 (76.1)	73 (81.1)	0.05	0.82
Y	959 (25.4)	25 (27.2)	72 (29.9)	58 (20.4)	75 (25.8)	93 (24.5)	120 (23.9)	189 (26.0)	217 (28.0)	93 (23.9)	17 (18.9)		
Used adequate clothing protection in wooded or tall grass areas§													
N	2,710 (78.4)	68 (72.3)	189 (77.5)	239 (82.7)	203 (69.8)	281 (76.0)	377 (75.6)	530 (73.3)	508 (65.9)	258 (66.5)	57 (64.8)	27.87	<0.001
Y	1,047 (30.3)	26 (27.7)	55 (22.5)	50 (17.3)	88 (30.2)	89 (24.1)	122 (24.5)	193 (26.7)	263 (34.1)	130 (33.5)	31 (35.2)		

*Case-patients were queried on risk factors and preventive behaviors in the 30 d before illness onset. A low percentage of case-patients provided responses to queries on risk factors (46%–64%) and preventive behaviors (48%–50%). †Cochran-Mantel-Haenszel test; p<0.05 represents a statistically significant relationship between risk factor/preventive behavior and increasing age group. ‡For activities in wooded or tall grass areas, case-patients were asked about specific activities such as hiking, camping, or hunting. §Adequate clothing included long sleeves, long pants, and covered shoes.

Relatively few case-patients who self-identified as male or female reported specific preventive behaviors while outdoors in wooded or tall grass areas (Table 3). Among respondents to specific preventive behavior questions, 1,265/3,915 (32.3%) of case-patients reported checking themselves for ticks, 1,046/3,750 (27.9%) reported wearing adequate clothing, and 958/3,763 (25.5%) reported using insect repellents in regions known to be habitats for ticks. Practicing preventive behaviors did not differ by sex (p>0.05). As age increased, more case-patients reported wearing adequate protective clothing while in tick habitats (n = 3,757, df = 1, χ^2^ = 27.9; p<0.001) (Table 4). 

## Discussion 

Lyme disease continues to spread in Ontario; incidence rates increased 3-fold during the 2015–2022 study period. Incidence rates increased in a biennial pattern, similar to nationwide trends in Canada during 2016–2022 and the United States during 2004–2021 (*1*,*5*,*22*). The biennial trend likely reflects changes in the abundance of *B*. *burgdorferi*–infected nymph-stage blacklegged ticks; that stage is responsible for most summertime infections from ticks. Abundance of ticks is governed by ecologic variables such as temperature, precipitation, host abundance, tick distribution, and human exposure levels (*23*,*24*). Male case-patients, children <10 years of age, and adults >59 years of age were the groups with the highest Lyme disease illness rates in Ontario; however, exposures indicative of risk or protective behaviors depended on demographics. This information presents an opportunity to develop educational resources targeted to persons less likely to wear appropriate clothing or repellents while in tick habitats. Our research constitutes an initial step toward identifying behavioral, ecologic, and biomedical factors contributing to sex- and age-specific risks of Lyme disease. 

Male case-patients, children 5–9 years of age, and adults 50–79 years of age experienced higher Lyme disease incidence than female case-patients and other age groups, similar to trends reported elsewhere (*4*,*13*,*25*–*27*). In the United States in 2021, of persons taking part in outdoor recreational activities (e.g., hiking, camping, fishing), 54% (95.5 million/176.7 million) were male, potentially disproportionately exposing males to blacklegged ticks (*28*). Data from Ontario during 2005–2014 indicated 50% of case-patients were male, compared with 56% in our study, with a relatively higher incidence among those 5–9 and 50–74 years of age (*25*). Similarly, in Canada during 2009–2019, a total of 57% of case-patients were male, and the highest incidence was among those 5–14 and 50–84 years of age (*5*). In the United States during 2012–2016, the proportion of cases peaked among those 5–9 and 45–70 years of age; the proportion of male case-patients with Lyme disease and the median case-patient age increased over the broader study period, 1992–2016 (*14*). Lyme disease incidence trends likely reflect sex- and age-specific behavioral differences; however, case trends follow in part from susceptibility of individual persons to clinical disease and subsequent likelihood of case detection and the population structure in areas of highest incidence (*14*). Furthermore, the higher incidence among young children might reflect increased healthcare-seeking behavior by parents and children who spend more time outdoors in potential tick habitats. Trends in found ticks submitted by healthcare providers and the public (i.e., passive tick surveillance) parallel the epidemiologic picture of Lyme disease (*29*–*31*). Those epidemiologic trends indicating possible associations between increased healthcare-seeking behaviors and tick submissions provide rationale for developing targeted public health messaging for specific groups, such as young children (including parents and caregivers), older adults, and persons who take part in outdoor activities. Additional research to assess the most effective public health messaging for preventing Lyme disease in these groups is warranted. 

The evidence is inconsistent for increased risk for *B. burgdorferi* infection among owners of companion animals, despite companion animal owners having an increased risk for tick exposure (*32*,*33*). Because our analyses did not adjust for potential confounding or effect modification by other variables, factors related to ownership of companion animals with access to the outdoors or living in rural areas might have interacted with one another to influence reported Lyme disease incidence. For example, companion animals and their owners in rural areas might be more likely to share exposures to more blacklegged tick habitats than those in urban or suburban areas (*34*–*36*). Male case-patients and children <10 years of age engaged more often in outdoor activities with the potential for exposure to blacklegged ticks. The increased risk of Lyme disease for males is evident not only for other vectorborne diseases (e.g., West Nile virus infection) but is also associated with poor health-seeking behaviors, childhood obesity, and poor nutrition (*37*–*40*). Previous behavioral surveys and seroprevalence surveys in North America have found that female and older participants were more often knowledgeable than male participants and younger children about Lyme disease, risk factors for infection, and adopting preventive behaviors (e.g., use of repellents, wearing protective clothing) (*17*,*41*,*42*). Similar to our finding that female case-patients were more likely to report practicing preventive behaviors than male case-patients, in a study from the Estrie region of Québec, female participants were more aware of Lyme disease and more concerned about infection than male participants; Lyme disease awareness was also higher among persons >35 years compared with those <25 years of age (*17*). Female participants used tick repellents and performed tick checks more often than male participants, although male participants showered after outdoor activities more often than female participants (*17*). In contrast to the literature, aside from some older case-patients reporting wearing adequate clothing in tick habitats, we identified no additional sex- or age-related differences in use of preventive measures. Although provincial questionnaires aimed to standardize case interviewing, not all case-patients were asked about all risks and preventive behaviors (i.e., questions about some variables were reported as not asked), potentially concealing differences associated with age and sex. Readers should interpret our results with caution, as a low percentage of case-patients provided responses to queries on risk factors (46%–64%) and preventive behaviors (48%–50%); the lack of responses was likely because of lack of prompting by the interviewer, rather than declining to answer. Investigators might benefit from training and reminders to ensure consistency and completeness in case interviewing. 

The most common voluntarily reported locations for exposure among case-patients in Ontario occurred in the peridomestic environment, either at a secondary (46%) or primary residence (31%); however, only 53% of case-patients reported information on exposure location. Because peridomestic and areawide tick and rodent control are yet to show efficacy in reducing Lyme disease incidence, alternative and complementary methods should be explored (*43*,*44*). Focused public health messaging for those at higher risk of developing Lyme disease is vital, but the incidence of peridomestic Lyme disease warrants focused public health guidance on preventing tick exposures through activities around the home (e.g., gardening). 

Among limitations to our study, aside from those already discussed, true incidence of Lyme disease is subject to underreporting, which can be caused by multiple factors, such as low disease awareness, poor healthcare-seeking behaviors, preferential ascertainment of severe disease, limitations of serology during early infection, and reporting practices (*45*,*46*). Underreporting of Lyme disease in Ontario likely occurred during the study; however, we do not expect any difference in demographics and exposures of patients between reported and unreported cases. Second, the Ontario provincial electronic reportable disease system does not provide closed-ended questions on Lyme disease exposure locations (i.e., secondary residence), and completeness and entry of case exposure information relied on the data entry practices of PHUs and individual investigators. Consequently, some cases might not have a reported exposure location, and others might have multiple reported exposure locations. In future enhancements to the system, developers should consider adding specific questions regarding exposure locations. Third, there was potential for misclassification of exposure in our study because not all reported exposures contributed to *B*. *burgdorferi* transmission and additional contributing exposures might not have been reported; the exact location of tick acquisition was not identifiable and had to be inferred. Fourth, there is potential for recall bias among study case-patients, because those who found ticks on themselves might have spent more time thinking about whether they engaged in higher risk activities or took preventive measures. Fifth, case-patients were not asked specifically about use of other preventive measures (e.g., putting clothes in the dryer after being in tick habitats, limiting tick habitats around the home, use of tick prevention on pets); therefore, data are likely incomplete on the use of other preventive measures. 

Despite limitations, the data we collected provided an opportunity to identify factors responsible for differences in observed sex- and age-specific exposures. Standardized epidemiologic studies (e.g., case-control) will improve the ability to identify the factors underlying the demographic patterns seen in Lyme disease surveillance. A proposal in Quebec aims to prioritize prevention efforts using a One Health approach that includes integrating behavioral (preventive) methods and ecologic (tick surveillance) risks into risk mapping (*47*). Protection motivation theory states that a person’s use of protective measures increases along with the perceived seriousness of tick bites and Lyme disease (*48*). Protection motivation theory has the potential to predict the demographic and environmental factors that would increase the likelihood of the public using preventive measures; interventions to address those factors include messaging targeted to at-risk subpopulations that currently do not perceive tick bites and Lyme disease as serious threats to their health. A 2022 systematic review (*49*) found the effectiveness of various personal protection measures inconsistent and no single method effectively prevented Lyme disease. The success of prevention depends on effective public health actions that include passive and active surveillance, health messaging on personal preventive measures, and increasing Lyme disease awareness (e.g., risks posed by tick bites, seriousness of disease, risk mapping, epidemiology). 

Using disease data in Ontario reported during 2015–2022, we found higher incidence of Lyme disease among male case-patients, children <10 years of age, and adults >59 years of age. In general, male case-patients were more likely than female to report finding a tick on themselves, and older age groups reported wearing adequate protective clothing while in blacklegged tick habitats more often than younger age groups. Until populationwide prevention methods (e.g., vaccines, areawide tick and rodent control) effective at reducing Lyme disease incidence become available, education and guidance should focus on behavioral change and personal protection targeted to specific subpopulations at high risk for disease. 

Appendix Additional information on differences by age and sex in Lyme disease health-related behaviors in Ontario, Canada, 2015–2022
