# Supplementary material for: Age- and Sex-Specific Differences in Lyme Disease Health-Related Behaviors, Ontario, Canada, 2015–2022
Source: Emerg Infect Dis. 2024 Oct;30(10):2006–15. doi: 10.3201/eid3010.240191 (PMC11431918; doi:10.3201/eid3010.240191)
Supplement: Appendix — Additional information on differences by age and sex in Lyme disease health-related behaviors in Ontario, Canada, 2015–2022 [file 24-0191-Techapp-s1.pdf]

Article DOI: https://doi.org/10.3201/eid3010.240191

EID cannot ensure accessibility for supplementary materials supplied by authors. Readers who have difficulty accessing supplementary content should contact the authors for assistance.

Age- and Sex-Specific Differences in Lyme Disease Health-Related Behaviors, Ontario, Canada, 2015–2022

Appendix

Appendix Table. Case definitions used in study

Confirmed	Probable
Clinician-confirmed *Erythema migrans* >5 cm in diameter with a history of residence in, or visit to, a Lyme disease endemic area or risk area<br>OR<br>Clinical evidence of Lyme disease with laboratory confirmation by PCR or culture<br>OR<br>Clinical evidence of Lyme disease with laboratory support by serologic methods, and a history of residence in, or visit to, an endemic area or risk area	Clinical evidence of Lyme disease with laboratory support by serologic methods, but with no history of residence in, or visit to an endemic area or risk area<br>OR<br>Clinician-confirmed *Erythema migrans* >5 cm in diameter but with no history of residence in, or visit to an endemic area or risk area

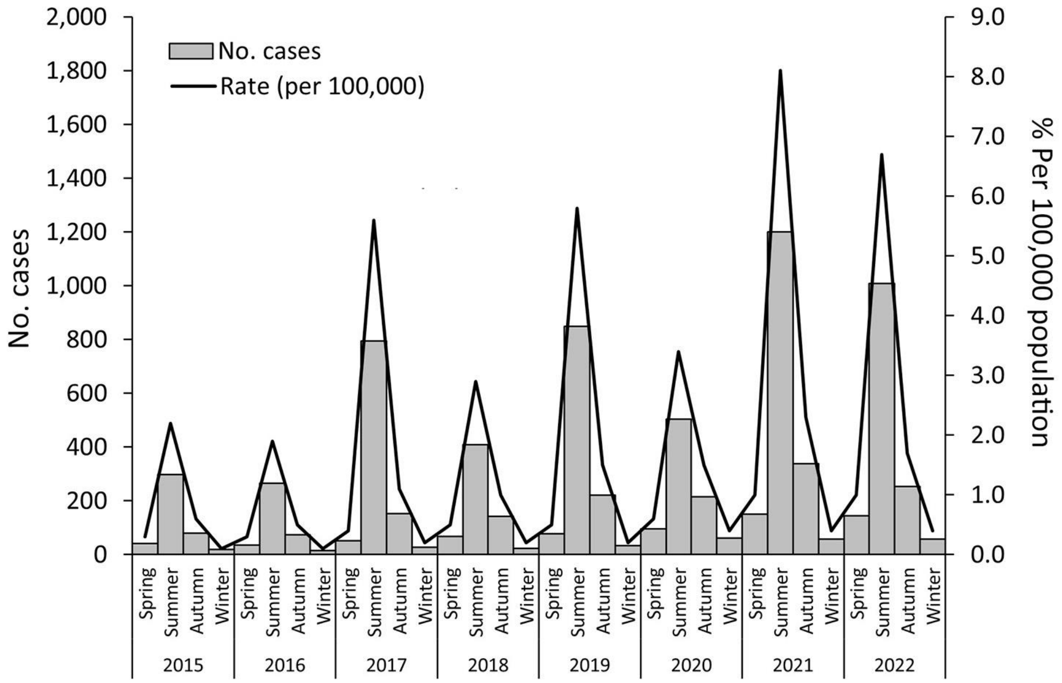

Appendix Figure 1. Seasonality of Lyme disease in Ontario, Canada (2015–2022). Denominator used in rate calculation includes annual population estimates. Seasons. Spring: March–May, Summer: June–August; Autumn: September–November; Winter: December–February.

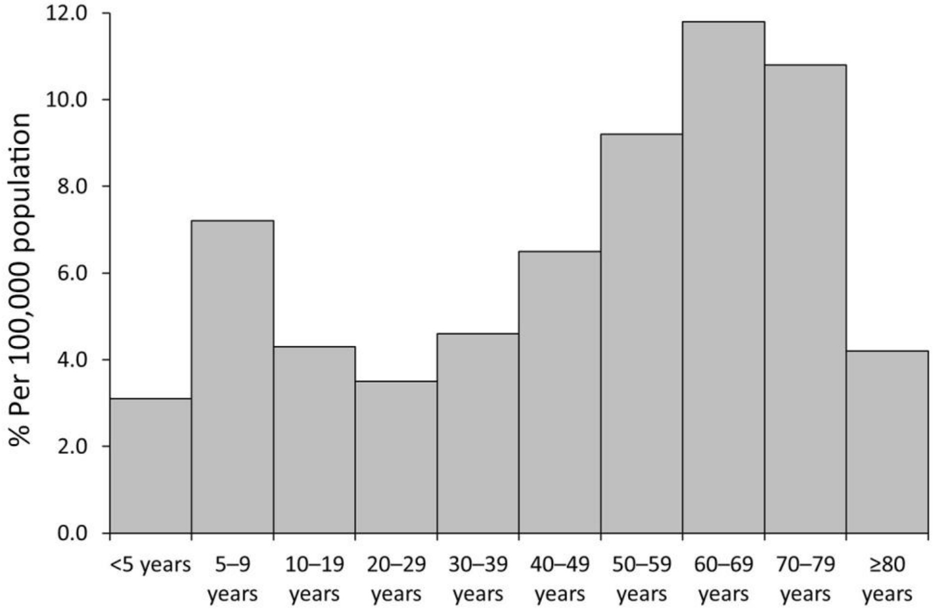

Appendix Figure 2. Average annual rate per 100,000 population of Lyme disease in Ontario, Canada (2015–2022) by age group.
